# Development and Application of an Alert System to Detect Cases of Food Poisoning in Japan

**DOI:** 10.1371/journal.pone.0156395

**Published:** 2016-05-27

**Authors:** Akie Maeyashiki, Manabu Akahane, Hiroaki Sugiura, Yasushi Ohkusa, Nobuhiko Okabe, Tomoaki Imamura

**Affiliations:** 1 Department of Public Health, Health Management and Policy, Nara Medical University School of Medicine, Kashihara, Japan; 2 National Institute of Infectious Diseases, Infectious Disease Surveillance Center, Tokyo, Japan; 3 Kawasaki City Public Health Institute for Public Health, Kawasaki, Japan; National Taiwan University, TAIWAN

## Abstract

Recent public health concerns regarding commercial food products have increased the need to develop an automated method to detect food product-related health events. We developed and verified a method for the early detection of potentially harmful events caused by commercial food products. We collected data from daily internet-based questionnaires examining the presence or absence of symptoms and information about food purchased by the respondents. Using these data, we developed a method to detect possible health concerns regarding commercialized food products. To achieve this, we combined the signal detection method used in the reporting system of adverse effects of pharmaceutical products and the Early Aberration Reporting System (EARS) used by the United States Centers for Disease Control. Whiteleg shrimp (*Litopenaeus vannamei*), which had odds ratio and Odds(−) of 8.99 and 4.13, respectively, was identified as a possible causative food product for diarrhea and vomiting. In conclusion, this study demonstrated that food distributors can implement post-marketing monitoring of the safety of food products purchased via the internet.

## Introduction

Hygienic management of food products is particularly important to humans. In the United States (US), the National Strategy for Biosurveillance was implemented in 2012 to enable the early detection of health concerns related to food [1]. The Foodborne Diseases Active Surveillance Network (known as FoodNet), an emerging infection program, has implemented active epidemiological surveys to detect emerging infections; this has been done in collaboration with the US Centers for Disease Control and Prevention (CDC) [2].

To detect and manage food poisoning at an early stage, it is necessary to monitor the causative pathogen and the contaminated food. The incidence and distribution of food poisoning cases have increased in recent years because foods produced in one location are now sold in larger areas because of the development of efficient distribution systems. For example, in 2008 in Japan, there were several cases of food poisoning caused by frozen dumplings that had been produced in China and were contaminated with methamidophos, an organophosphorus pesticide [3]. In 2013, there was a case where a worker intentionally added malathion, an agricultural chemical, into frozen food during the production process. These cases raised considerable attention concerning the safety of foods consumed by the general public. For the early detection of health concerns of mass-produced foods, it is necessary to implement a crisis management system together with post-marketing monitoring (PMM) of food products.

Pharmaceutical products have much stricter controls than food products, and post-marketing surveillance (PMS) programs are routinely performed for pharmaceutical products. The Uppsala Monitoring Centre of the World Health Organization and regulatory authorities have developed pharmacovigilance databases for the early detection of potential adverse effects of pharmacological products [4]. In the Netherlands, the Netherlands Pharmacovigilance Centre Lareb maintains a self-reporting system to record the adverse effects of pharmacological products. At Lareb, a reporting odds ratio (ROR) method, which was initially developed by Meyboom et al., has been further developed and used to monitor safety issues since 2000 [5–10].

PMS not only involves the early detection of adverse effects, but it also includes the safety management of vaccines by PMM [11] as well as the monitoring of erroneous use and abuse of drugs [12]. In 2010 in Japan, Ooba et al. used Japanese self-reported data to investigate the effects of under-reporting in signal detection systems based on the ROR method [13].

Unlike food products, pharmacological products are individual products, and if an adverse effect occurs in Japan, the patient should notify the prescribing doctor, who should then notify the Pharmaceutical and Medical Devices Agency. Regarding food products, Hepburn et al. summarized the reports on PMS of new food products in 2008 [14].

Several factors hinder the ability to implement post-marketing health surveys of food products. In particular, the distribution routes of food products are complex. It is also difficult to identify who purchases individual food products. For these reasons, no methods have been developed to perform PMM of food products. To address this issue, we examined whether a method that is already used clinically to detect adverse effects of pharmacological products can also be applied to the early detection of potentially harmful events related to post-marketing food products.

We have developed the Web-based Daily Questionnaire for Health (WDQH), a syndrome surveillance/early detection system, in which a daily survey records physical symptoms (health information) directly from community residents [15]. This system is useful for the detection of outbreaks of infections in the community and for performing health surveys of community residents in relation to environmental factors [16,17].

In the current study, we used a database that combines individual daily health information with a list of foods purchased by individual people. We developed a system that sends alerts to the users if specific symptoms are associated with specific foods. We also used this system to evaluate the practicability of a food PMM for the early detection of food-related issues.

## Methods

### Ethics statement

This study was approved by the Ethical Committee of Nara Medical University (approval number: 220). Written informed consent for each participant was obtained at the registration screening.

### Participants

The participants were customers and their families who purchased foods through the websites of Pal-system Tokyo Consumers’ Co-operative (Tokyo) and Osaka Izumi Co-operative Society (Osaka). The databases included 1002 households in Tokyo (3128 people, including family members) and 554 households in Osaka (1925 people, including family members).

### Implementation of the health survey

Health surveys were conducted according to the method of WDQH [15]. We focused on two of the health survey items, diarrhea and vomiting, because they are strongly associated with food poisoning. With support of the Japanese Consumers’ Co-operative Union (CO-OP), the surveys were implemented in two cities, Tokyo and Osaka, in Japan between January 20 and April 30, 2011.

Subjects who consented to participate in the survey were registered and agreed to provide the dates that food products were purchased during the survey period. For the health survey, which was accessed online using a personal password, the registered subjects received daily remainder e-mails to answer questions regarding the presence or absence of symptoms in themselves and/or their family members. All participants were given a reward, equivalent to JPY 500 (approximately US$5), at the end of the survey period (S1 and S2 Files).

### Food-purchasing survey

Data on purchased foods were obtained from CO-OP every 2 weeks. As a baseline, we obtained data on foods purchased for 4 weeks before the start of the health surveys. The data included the member identity code (ID), date of the order, list of foods ordered, Japanese Article Number code, stock keeping unit, and number of items ordered. The data were anonymized in a similar manner to the personal information.

### Data management

Health information and food purchase data were linked with the subject’s ID. In the data analyses, we needed to consider two limitations of the database. First, the database did not record which family member(s) ate a specific food. Thus, the survey was performed for the family unit, and if one family member reported a specific symptom on one day, the family was regarded as being a symptomatic household. Second, although the food delivery date was recorded, the date of consumption was not. To overcome this limitation, the period of food intake was defined as the time between delivery to the expiration date of the food product, or a maximum of 30 days for frozen food products.

### Analytical methods

All statistical analyses were conducted using Microsoft Excel and Microsoft Access, as described in the following sections.

#### Step 1: EARS screening

We used the CDC EARS [18] to identify dates on which the number of reports of diarrhea and/or vomiting increased rapidly as possible cases of food poisoning. Unlike vomiting, there is a constant number of subjects with chronic symptomatic diarrhea that is independent of the consumed food products. The daily mean proportion of all respondents who reported each symptom was 2% for diarrhea and 1% for vomiting [15]. Thus, it is possible that symptoms of food poisoning may have been overlooked for foods sold in small quantities.

To increase the sensitivity for the detection of diarrhea, the parent group of foods was divided into 20 subgroups, and the EARS was separately applied to vomiting and diarrhea groups as well as 20 diarrhea subgroups. For the 20 subgroups, we included the condition that ≥2.5% of households needed to report the symptom for detecting a possible association between the food subgroup and the symptom. We also set the condition that at least three households needed to purchase a food that was possibly and temporally related to an episode of food poisoning.

#### Step 2: Screening using the signal detection method

We first sought to detect possible signals of food poisoning and symptoms by extracting data obtained in Step 1. The World Health Organization defines a signal as information that requires further investigation and is accompanied by a possible causal relationship between a pharmacological product and adverse events that is currently unknown or has been incompletely investigated [19]. In this study, we replaced pharmacological products with food products in the signal detection process.

For signal detection, we prepared a 2 × 2 contingency table, with food products in the rows and the absence/presence of symptoms in the columns of the table (Table 1). To calculate the frequency of events, the date of signal detection by EARS was designated as the starting point, and data were collected in 1-week units for the previous 4 weeks. If the suspected food was consumed on ≥1 day(s) in a given week, that week was classified as positive exposure to the food product. If a specific symptom was reported on ≥1 day in a given week, that week was regarded as symptomatically positive.

**Table 1:** 2 × 2 Contingency Table for Reporting Adverse Events.

	Specific adverse event (diarrhea or vomiting)	All other adverse events	Total
Specific food	n_11_	n_12_	n_1+_
All other foods	n_21_	n_22_	n_2+_
Total	n_+1_	n_+2_	n_++_

We used the ROR method, which was introduced by the Netherlands Pharmacovigilance Centre Lareb [6–10] for signal detection. The ROR is an ordinary odds ratio (OR) and the lower limit value (threshold value or Odds(−)) of the 95% confident interval is used as the standard for signal detection [8,10,20,21]. The Odds(−) in PMS of food products was calculated using the equations presented below [10]. Using these equations, we calculated the OR for cases with symptoms of food poisoning attributable to a specific food product (n_11_/n_21_) and for cases without symptoms after consuming other food products (n_12_/n_22_). We then calculated the ORs for cases without symptoms who consumed other food products and cases with symptoms who consumed the specific food product. Next, we calculated the Odds(−); Odds(−) >1 was considered a positive signal.

A value of n_11_ = 0 indicated no combination of a specific food product and symptom. A value of n_22_ = 0 indicated no combination between other food products and other symptoms, including the absence of symptoms. Those cases are excluded from analyses. A value of n_12_ = 0 indicated no combination of a specific food product and a specific reported symptom. A value of n_21_ = 0 indicated no combination between other food products and the reported symptoms. For these two events, the value 0.5 was added to each cell in the contingency table.

Odds(−)=Odds/exp(1.96SE)

$$Odds\text{=}\frac{n_{11}/n_{12}}{n_{21}/n_{22}}$$

$$SE\text{=}\sqrt{\left(1/n_{11}+1/n_{12}+1/n_{21}+1/n_{22}\right)},$$

$$ROR\text{ =}\frac{n_{11}/n_{21}}{n_{12}/n_{22}}\text{=}\frac{n_{11}n_{22}}{n_{12}n_{21}}$$

$$SE\text{ \{log(}ROR\text{)\} = }\sqrt{\frac{1}{n_{11}}+\frac{1}{n_{12}}+\frac{1}{n_{21}}+\frac{1}{n_{22}}}$$

95%CI=exp[log(ROR)±1.96×SE{log(ROR)}].

To screen for potential signals in Step 2, we used the ROR threshold value as the reference value. After identifying food products with Odds(−) with >1 and >3 reported cases (i.e., n_11_) [10], we ranked them according to Odds(−) values in descending order and selected those products with an Odds(−) value ranked in the top 10.

#### Step 3: Analysis of scatterplots

After ranking the 22 groups (vomiting and diarrhea groups and 20 diarrhea subgroups) identified in Step 2, we prepared a scatterplot of the possible causative foods by displaying the OR values for the food products ranked in the top 10. The scatterplot arranges the occurrence of each symptom per household according to the timing of food consumption relative to the symptom in a chronological order. The scatterplot was used to confirm the family structure of the affected household, age, presence or absence of simultaneous symptoms in the household, presence or absence of simultaneous diarrhea/vomiting, and correlation between the date of the symptom and time of consumption. To examine the possibility of a causal relationship between the food product and the reported symptom (Table 2), an epidemiologist with expert knowledge verified whether the scatterplot indicated an outbreak of food poisoning, with consideration of the patients’ daily symptoms. If there were sudden occurrences of symptoms or if there were other patients with symptoms in the same time of period from other families who had the same meal as multiple members in the family, we implemented further investigation, considering food poisoning. We considered sustained symptoms as noninfectious digestive symptoms.

**Table 2:** Data Used to Generate the Scatterplot.

Member ID	Sex/Age	Date (February)							
		10	11	12	13	14	15	16	17
137	M31								
	F31								
	F8					▲	▲		
	M4		△	△	●	△	△		
501	M47								
	F43								
	M12								
	M8				●	●	●	●	△
538	M56								
	F56	△				△	△	△	△
	F24					△			△

M: male, F: female; △: diarrhea only; ▲: vomiting only; ●: diarrhea and vomiting.

## Results

### Responses to the health survey

In total, 56,340 responses were obtained in Tokyo (174,173, including family members) and 33,596 responses were obtained in Osaka (114,881, including family members) during the index period.

### Purchase of foods

Overall, during the index period, 6212 different food products were purchased in Tokyo, and 5392 different food products were purchased in Osaka.

### Alert detection

#### Step 1: Screening by EARS

EARS was performed during the survey period for all the foods and the number of days on which diarrhea or vomiting were reported, and the results are shown in Table 3. The list of possible causative foods was refined by applying the signal detection threshold in Step 1 (Table 4). Possible causative foods were extracted, and 1.9–3.3% of the foods were associated with diarrhea, and 0.3–0.4% were associated with vomiting.

**Table 3:** Number of Days on which Symptoms were Detected using the Early Aberration Reporting System.

	Tokyo	Osaka
Diarrhea (all food products)	26	24
Diarrhea (20 subgroups of food products)	45	42
Vomiting (all food products)	19	16
Total number of food products purchased during the study period	6212	5392

**Table 4:** Number of Possible Causative Foods Identified in Step 1.

	Tokyo		Osaka	
	N	%	N	%
Diarrhea (all food products)	206	3.3	103	1.9
Diarrhea (20 subgroups of food products)	200	3.2	103	1.9
Vomiting (all food products)	24	0.4	15	0.3

#### Step 2: Screening of potential signals

The food products ranked in the top 10 using the Odds(−) determined in Step 1 are shown in Tables 5 and 6 for Tokyo and Osaka, respectively. Only alerts identified using the EARS on or after February 1 were included in the analysis.

**Table 5:** Odds Ratio Ranking of Food Products Associated with Diarrhea and Vomiting in Tokyo.

	No	Date of detection	Type of product	n_11_	n_++_	OR		Odds(−)	
Diarrhea	All food products								
	1	2011/02/10	Chinese-style hotchpotch noodles	11	3176	6.32		3.13	
	2	2011/04/13	Grilled pork innards	6	1766	6.05		2.38	
	3	2011/02/10	Dried whitebait	26	3176	3.56		2.27	
	4	2011/02/11	Pureed squash	7	3241	4.61		1.98	
	5	2011/04/28	Steamed meat bun	7	2781	4.42		1.92	
	6	2011/03/13	Seafood pilaf	22	3080	3.08		1.9	
	7	2011/02/10	Flatfish fillet	7	3176	4.34		1.88	
	8	2011/02/09	Dried boneless mackerel with sweet sake	14	2473	3.21		1.77	
	9	2011/04/25	Hamburger	12	2642	3.27		1.73	
	10	2011/04/11	Seasoning for boiled rice with chicken and burdocks	9	1720	3.6		1.71	
	20 subgroups of food products								
	1	2011/02/10	Chinese-style hotchpotch noodles	11	3176	6.32			3.13
	2	2011/04/13	Grilled pork innards	6	1766	6.05			2.38
	3	2011/03/12	Seasoning for boiled rice	12	3098	4.2			2.2
	4	2011/02/11	Pureed squash	7	3241	4.61			1.98
	5	2011/04/28	Steamed meat bun	7	2781	4.42			1.92
	6	2011/03/13	Seafood pilaf	22	3080	3.08			1.9
	7	2011/02/10	Flatfish fillet	7	3176	4.34			1.88
	8	2011/02/09	Dried boneless mackerel with sweet sake	14	2473	3.21			1.77
	9	2011/02/03	Noodles with deep-fried tofu	10	2369	3.57			1.77
	10	2011/02/20	Custard pudding	13	3244	3.08			1.67
Vomiting	All food products								
	1	2011/03/12	Frozen fish-shaped pancake stuffed with custard	4	3098		6.2		2.13
	2	2011/03/12	Yogurt	11	3098		3.34		1.71
	3	2011/03/06	Rice	7	3134		2.9		1.29
	4	2011/02/16	Salted mackerel fillet	17	3271		2.13		1.23
	5	2011/04/11	Milk	9	1720		2.71		1.19
	6	20110206	Cut spinach (frozen)	9	2459		2.32		1.11
	7	2011/04/21	*Natto* (fermented soybeans)	10	2444		2.37		1.08
	8	2011/02/07	Pork sausage	16	2475		1.86		1.02
	9	–	Not detected	–	–		**–**		–
	10	–	Not detected	–	–		–		–

Dates are given as the year/month/day

OR: odds ratio.

**Table 6:** Odds Ratio Ranking of Food Products Associated with Diarrhea and Vomiting in Osaka.

	No	Date of detection	Type of product	n_11_	n_++_		OR	Odds(−)
Diarrhea	All food products							
	1	2011/02/12	Whiteleg shrimp	10	1818		8.99	4.13
	2	2011/02/10	Salted dried kelp	4	1763		7.39	2.3
	3	2011/03/12	Tofu	40	1826		2.91	1.94
	4	2011/02/26	Japanese-style confection	5	1851		5.43	1.92
	5	2011/03/10	Kelp with *shiso*	7	1834		4.12	1.76
	6	2011/02/20	Vegetable juice	5	1852		4.78	1.73
	7	2011/04/03	Green bean sprouts	14	1081		3.14	1.65
	8	2011/02/26	Burdock salad (with mayonnaise)	18	1851		2.79	1.63
	9	2011/02/15	Fried rice cake (rice crackers)	7	1837		3.6	1.55
	10	2011/02/15	Tomato ketchup	13	1837		2.87	1.54
	20 subgroups of food products							
	1	2011/02/12	Whiteleg shrimp	10		1818	8.99	4.13
	2	2011/02/27	Pasta sauce (tomato and basil)	6		1852	7.62	2.81
	3	2011/03/12	Tofu	40		1826	2.91	1.94
	4	2011/02/26	Japanese-style confection	5		1851	5.43	1.92
	5	2011/02/28	Burdock salad (with mayonnaise)	20		1850	3.11	1.85
	6	2011/03/10	Kelp with *shiso*	7		1834	4.12	1.76
	7	2011/02/20	Vegetable juice	5		1852	4.78	1.73
	8	2011/04/03	Green bean sprouts	14		1081	3.14	1.65
	9	2011/02/18	Tomato ketchup	14		1853	2.9	1.59
	10	2011/02/28	Cooked pork (with miso)	9		1850	3.31	1.57
Vomiting	All food products							
	1	2011/02/10	Canned tuna	16		1763	3.48	1.8
	2	2011/02/10	Whole tomatoes	5		1763	4.66	1.75
	3	2011/02/10	Tomato ketchup	5		1763	3.69	1.4
	4	2011/02/10	Mayonnaise	5		1763	3.53	1.34
	5	2011/02/26	Fried scallops	16		1851	1.92	1.07
	6	−	Not detected	−		−	−	−
	7	−	Not detected	−		−	−	−
	8	−	Not detected	−		−	−	−
	9	−	Not detected	−		−	−	−
	10	−	Not detected	−		−	−	−

Dates are given as the year/month/day

OR: odds ratio.

In Tokyo, the three food products with the highest Odds(−) for diarrhea were noodles, grilled pork innards, and dried whitebait. Among the 20 subgroups of food products, seasoning for boiled rice was associated with diarrhea. The three food products with the highest Odds(−) for vomiting were yogurt, rice, and frozen fish-shaped pancakes stuffed with custard (Table 5).

In Osaka, the three food products with the highest Odds(−) for diarrhea were whiteleg shrimp, salted dried kelp, and tofu. Pasta sauce was also found to be associated with diarrhea as one of the 20 subgroups of food products. The three food products with the highest Odds(−) for vomiting were canned tuna, whole tomatoes, and tomato ketchup (Table 6).

#### Step 3: Inspection of the scatterplots

Scatterplots showing the associations between the food products ranked in the top 10 using Odds(−) in Steps 1 and 2 were developed to visualize the timing of symptoms in each household. These scatterplots revealed that three symptomatic households consumed whiteleg shrimp (*Litopenaeus vannamei*) on the same day and reported multiple episodes of diarrhea within the household. Because the OR and Odds(−) values for whiteleg shrimp are very high (8.99 and 4.13, respectively), we considered that it had a possible causal relationship to food poisoning and hence issued an alert.

Considering this possible causative relationship, we tested this food product for bacterial contamination, and microbiological tests revealed the presence of *Vibrio parahaemolyticus* with a most probable number of 23/g. We also surveyed all households who purchased this food product during the survey period and confirmed that there were no additional episodes.

## Discussion

In this study, we conducted analyses of a large number of food products to establish and validate a PMM for food products, with the goal of early detection of health concerns (e.g., food poisoning) related to specific food products. To achieve this, we collaborated with two companies that sell food products via the internet to the general public and obtained data on the food products purchased by the participants.

In 2008, Hepburn et al. described a food PMM for artificial cholesterol (Olestra), artificial sweetener (aspartame), and StarLink maize (genetically modified food) [14]. However, they only investigated some specific foods that were not linked to symptoms of food poisoning. Furthermore, although they proposed a method for collecting data on household food purchases, they only presented the purchase records. To our knowledge, there are no previous reports of prospective food PMMs in which researchers tried to link large numbers of food products with specific symptoms.

Because of the widespread food poisoning caused by frozen dumplings made in China in 2008, initiatives aimed at the early detection of health concerns of post-marketing food products and preventing such concerns are being implemented in Japan [3]. In response to the food poisoning event, the Ministry of Health, Labour and Welfare established the Office of Foodborne Disease Surveillance. Its mandate is to conduct retrospective studies of information obtained from autonomous communities if food poisoning affects ≥ 50 people or if the cases are widely distributed. However, this approach only includes actual cases, and the Office of Foodborne Disease Surveillance cannot make comparisons with control groups. To address this issue, we performed more detailed analyses in subjects with or without daily symptoms as recorded using the WDQH [15]; moreover, we analyzed data regarding food products purchased by the subjects during the index period.

The EARS used in Step 1 is suitable for short-term symptom surveillance and does not require long-term data. In PMM of a single food product, Newkirk et al. conducted the EARS with data collected by the CDC (1990–2006) to determine a potential food defense index using food poisoning data for milk and verified this index in 2007 [22].

Using the results of the EARS and symptoms reported on a daily basis by the subjects, we were able to determine the dates on which the number of episodes of vomiting and diarrhea increased rapidly in association with a specific food product [18]. In the next step, we selected all events occurring in at least three households who purchased the relevant food product. The frequency of possible causative food products to be associated with diarrhea was 1.9% and 3.3% and that of vomiting was 0.3% and 0.4%, in Osaka and Tokyo, respectively. There were a constant number of subjects with chronic symptoms of diarrhea [15].

We also conducted further analyses to refine the overall group of food products and found that it was necessary to identify food products that were associated with symptoms, even if they were purchased in small quantities. This suggests that less-frequently purchased foods may be overlooked by the EARS, and it is sometimes difficult to detect symptoms caused by a specific food product, considering the vast number of different food products purchased in the index period. Although the sensitivity of the signal is particularly important in EARS-based analyses, false-positive results are possible in analyses involving small numbers of subjects. Thus, it was necessary to combine the EARS with other methods. To address this issue, we classified the food products into 20 subgroups for the analyses.

Using the EARS, we were able to identify possible dates on which symptoms of food poisoning occurred with a high frequency of symptoms. We were able to execute the PMM for food products by refining the list of possible causative food products for food poisoning to ≤5% of all purchased food products.

In the signal detection step (i.e., Step 2), we constructed a 2 × 2 table. This included a control group of symptomless subjects who did not purchase the specific food products. Using the screening results obtained by applying the ROR signal detection standard [8,10,20,21], we searched for possible signals of causative food products for food poisoning that were retrieved in Step 1.

After ranking the food products according to their Odds(−) for vomiting/diarrhea (Tables 5 and 6), we identified which food products were associated with large numbers of symptomatic subjects among the households that purchased the specific food product compared with households that did not purchase the food product. The food product with the highest OR was whiteleg shrimp, which indicates that the method used for signal detection of causative food products for food poisoning is practicable.

In 2006, the Wisconsin State Laboratory of Hygiene conducted a comparative control study of 86 cases and 49 controls in a community affected by an outbreak of *Escherichia coli* 0157. They revealed that spinach was the causative food with an OR of 82.1 [23]. That study used the PulseNet database, in which relevant organizations share information regarding food-borne infections to rapidly detect any food-related infections and determine the source. Although our system has similar aims, our approach also allowed us to perform follow-up surveys of affected cases.

After identifying food products potentially associated with food poisoning, Step 3 involved drawing scatterplots to visually investigate the relationship between the timing of events and determine whether a final alert should be issued. Statistical relationships do not necessarily indicate a causative relationship, and they may reflect some bias in the data and factors introduced by the investigator. Thus, before concluding that the observed signal is indicative of a causative relationship, the relationship must be considered in the context of our scientific knowledge and experience. Thus, a completely warranted medical evaluation helps achieve a scientific consensus [20,24].

In the present study, whiteleg shrimp had the highest odds for food poisoning, and we issued a final alert to raise awareness that it was a possible causative agent for food poisoning. We also obtained samples of the remaining food product to conduct microbiological tests for pathogenic bacteria and hence verify the causative relationship between the food product and symptoms of food poisoning.

To our knowledge, there have been no prospective PMM of food products in which the households that purchased specific food products were linked with the symptoms of food poisoning (i.e., diarrhea and vomiting) in a family member. Using this approach, we confirmed that PMM could be implemented for food products purchased from an internet-based distributor, and food products associated with an adverse outcome were identified using a three-step screening system.

Regarding the limitations of this study, it is possible that food products purchased from other retailers might be eaten simultaneously. Additionally, all symptoms recorded in the health surveys were self-reported, and any underlying diseases in the subjects might influence their reported symptoms.

To specifically evaluate the relationship between the possible causative food products detected in this study and health concerns, it will be necessary to conduct more detailed analyses of historical data, including the subjects’ individual health conditions, foods consumed, and seasonal eating habits; confirm the presence or absence of the purchase food claims by purchasers; and test food samples before shipment. Furthermore, to increase the methodological accuracy, we should consider implementing PMM, in which multivariable analyses are conducted where sex, age, consumption of the specific food product, and region are included as explanatory variables and the presence/absence of symptoms, as the dependent variable. The PMM should also include a system to alert for residual error within three standard deviations of the actual measured value. From a practical perspective, it will be necessary to improve the methodology to allow real-time alerting of possible outbreaks.

## Conclusion

The present study demonstrated the feasibility of implementing PMS of food products purchased via the internet. We combined the EARS with an endemic detection algorithm originally developed by the US CDC with a signal detection method that is already used for the early detection of adverse effects of pharmacological products. We believe that the PMM method used in this study represents a quantitative and qualitative method for detecting potential health issues of food products. The analytical approach used in this study could also be applied to chemically related food poisoning, not just microbial food poisoning. Finally, we wish to highlight the importance of early detection of symptoms of food poisoning that are related to a specific food product to prevent widespread cases of food poisoning.

## Supporting Information

S1 File The questionnaire research items. (XLS)

S2 File The questionnaire/survey which includes research outlines, explanations to participants. (DOCX)
