# Supplementary material for: Development and Application of an Alert System to Detect Cases of Food Poisoning in Japan
Source: PLoS One. 2016 May 27;11(5):e0156395. doi: 10.1371/journal.pone.0156395 (PMC4883778; doi:10.1371/journal.pone.0156395)
Supplement: S2 File — (DOCX) [file pone.0156395.s002.docx]

Request for Cooperation in Web-based Health Survey

We would like to request your kind cooperation in a “Web-based Health Survey.”

Over the years, the Japanese Consumers' Co-operative Union (JCCU) has conducted a “Web-based Health Survey” with the cooperation of the member Co-ops as a social experiment to create a mechanism for the “early detection of signs of a health crisis in the regions” and the “early detection of adverse health effects from particular foods.” This is a research carried out in cooperation with experts and an initiative that facilitates the early detection of infectious diseases, such as influenza, as well as mass outbreaks of food poisoning in initial stages. The JCCU is requesting the cooperation of members of the Co-op in registering as monitors for the study. We plan to collect and analyze data for each region using the information from the web-based reports on your daily health. We are grateful for the kind understanding and cooperation of all members of the Co-op for this survey. The survey will last for about three months. Members that register for the survey will receive 500 points. Click on “More details” for more information.

<Overview of Survey>

・Application period: Please register by January 18, 2011

・Survey period: January 20 to April 30, 2011

・Survey target: Co-op members living in the Tokyo Metropolitan area
(first 1,000 people)

・Survey items: Questionnaire on health conditions and symptoms when responding

・Response intervals: Respondents may select from “every day,” “every other day,” or “every two days.”

・There will be one additional questionnaire after the health survey is finished (beginning of May).

・Remuneration: Respondents will receive 500 points. (Tabulated responses and analysis results will be displayed on the map and daily comments will be sent.) Depending on the type of response interval selected, respondents will receive an email with a link to the questionnaire every day, every other day, or every two days. The time required for the interview is about three minutes each time.

<Registration for Monitors>

Please register as a monitor if you would like to take part in the survey. Registration is a two-step process. First, we will ask if you would be able to answer a questionnaire about your daily health conditions or symptoms. Next, we will ask if you would agree to provide information on the types of products you purchase door-to-door, which will be used for post-marketing monitoring (Monitors do not need to enter information on products purchased.) Even if you do not agree to provide information on the types of products you purchase, your cooperation is appreciated in submitting responses about your daily health conditions or symptoms.

<Handling Personal Information>

To register as a monitor for the survey, you will need to enter your personal information, including email address, age, address (up until city name), and Co-op membership number. (Your 8-digit Co-op membership number is listed in your delivery statement.) The personal information that you have registered and information on products you purchased is strictly controlled and will not be used for any purpose other than for this study. Information on products purchased will be stored separately from your personal information, which means that it will not be possible to identify what person has purchased what products. This information and responses received via questionnaires will not be made public. We will not send advertising or solicitation messages to the email address that you have registered after the study.

<Inquiries about the Survey and Registration>

Secretariat of the Japanese Consumers' Co-operative Union: palsystem-survey@mri.co.jp

(Please fill in your membership number, name, and telephone number when contacting the JCCU.)

Confirmation of Agreement

This is to confirm your agreement to register as a monitor for the web-based survey on your health conditions or symptoms. Please read the following. If you agree, please click “I agree” at the bottom of the screen to proceed to the next step.

The questionnaire includes the following items: items related to daily health and symptoms (Aggregate results will be used to analyze infections and allergic diseases, etc.). Questionnaire items include the presence of low-grade and high-grade fevers, runny nose, coughing, diarrhea, vomiting, seizures, itchy eyes, rashes, joint pain, sneezing, itchy skin, dry/cracked hands, insomnia, headaches, sore throat, stomach or abdominal pains, and if a person has been diagnosed with influenza or infectious gastroenteritis.

Aggregate results will be displayed on a map every day with comments that can also be viewed by monitors. The health situation of neighboring areas can also be viewed at a glance, which helps in the preparation for potential health crises.

I agree I do not agree

(People that agree to take part in the heath survey, but do not agree to provide information on products purchased, should still select “I agree” on this screen and go to the next screen where selections are available for the submission of information on products purchased.)

Confirmation of Agreement

This is to confirm your agreement to provide information on the types of products you purchased for use in post-marketing monitoring (PMM). Please read the following. If you agree, please click “I agree” at the bottom of the screen to proceed to the next step. (If you want to participate only in the health survey and do not want to provide information on the types of products you purchased, click “I do not agree” at the bottom of the screen.)

Information that you have agreed to provide on products purchased door-to-door (product list) will be used for post-marketing monitoring (PMM) of food products. Information that you provide on products purchased will only be for products during the survey period. Information will be sent from Izumi Co-op to JCCU, and monitors do not need to enter information themselves.

Post-marketing monitoring (PMM) of food products intends to survey if “respondents that have purchased a specific product (food) have experienced some type of health problem (diarrhea, vomiting, etc.),” and is considered to be a useful method in minimizing the occurrence and spread of health problems from food products. However, this method has not yet been established in other parts of the world because of the complexity of data collection and analysis. The objective of this social experiment is to collect basic data to explore problems that occur in the development of this method. This does not infer that there is a risk in the food products from the co-op that you purchase door-to-door.

Provision of product purchase information I agree I do not agree

Confirmation Screen

You register in the questionnaire survey on daily health status and symptoms and the provision of product purchase information.

Data Input Screen

Q1.　Do you or any of the people living with you have any of the following symptoms?

Symptoms: Fever, runny nose, cough, diarrhea, vomiting, stomach or abdominal pain, seizures, itchy eyes, rash, joint pain, headache, sore throat, sneezing, itchy skin, dry/cracked hands, insomnia

If you or any of the people living with you have been diagnosed with influenza or infectious gastroenteritis (norovirus, etc.), please check here. (Mandatory)

Q2.　Please list all of the symptoms of each of the people in your household that are ill. (Mandatory)

Please verify that all questions have been answered. If everything is correct, click the “Send” button.

This is the end of the question. Thank you for your cooperation.
